# EEG Source Imaging Indices of Cognitive Control Show Associations with Dopamine System Genes

**DOI:** 10.1007/s10548-017-0601-z

**Published:** 2017-12-08

**Authors:** G. McLoughlin, J. Palmer, S. Makeig, N. Bigdely-Shamlo, T. Banaschewski, M. Laucht, D. Brandeis

**Affiliations:** 1 MRC Social, Genetic and Developmental Psychiatry Centre, Institute of Psychiatry, Psychology and Neuroscience, King’s College London, PO80, London, UK; 2 Swartz Center for Computational Neuroscience, Institute for Neural Computation, University of California San Diego, La Jolla, CA USA; 3 Department of Child and Adolescent Psychiatry and Psychotherapy, Central Institute of Mental Health, Mannheim, Medical Faculty Mannheim / Heidelberg University, Mannheim, Germany; 4 Department of Psychology, University of Potsdam, Potsdam, Germany; 5 Department of Child and Adolescent Psychiatry and Psychotherapy, Psychiatric Hospital, University of Zürich, Zurich, Switzerland; 6 Zurich Center for Integrative Human Physiology, University of Zürich, Zurich, Switzerland; 7 Neuroscience Center Zurich, University and ETH Zürich, Zurich, Switzerland

**Keywords:** EEG, Genetics, DRD4, COMT, ICA, Measure projection

## Abstract

Cognitive or executive control is a critical mental ability, an important marker of mental illness, and among the most heritable of neurocognitive traits. Two candidate genes, catechol-*O*-methyltransferase (COMT) and DRD4, which both have a roles in the regulation of cortical dopamine, have been consistently associated with cognitive control. Here, we predicted that individuals with the COMT Met/Met allele would show improved response execution and inhibition as indexed by event-related potentials in a Go/NoGo task, while individuals with the DRD4 7-repeat allele would show impaired brain activity. We used independent component analysis (ICA) to separate brain source processes contributing to high-density EEG scalp signals recorded during the task. As expected, individuals with the DRD4 7-repeat polymorphism had reduced parietal P3 source and scalp responses to response (Go) compared to those without the 7-repeat. Contrary to our expectation, the COMT homozygous Met allele was associated with a smaller frontal P3 source and scalp response to response-inhibition (NoGo) stimuli, suggesting that while more dopamine in frontal cortical areas has advantages in some tasks, it may also compromise response inhibition function. An interaction effect emerged for P3 source responses to Go stimuli. These were reduced in those with both the 7-repeat DRD4 allele and either the COMT Val/Val or the Met/Met homozygous polymorphisms but not in those with the heterozygous Val/Met polymorphism. This epistatic interaction between DRD4 and COMT replicates findings that too little or too much dopamine impairs cognitive control. The anatomic and functional separated maximally independent cortical EEG sources proved more informative than scalp channel measures for genetic studies of brain function and thus better elucidate the complex mechanisms in psychiatric illness.

## Introduction

The global health burden of psychiatric illness (Whiteford et al. 2013) and its serious consequences for the individual and society (Organization 2014) have encouraged recent efforts to improve understanding of the etiology and pathophysiology of mental disorders. More precise phenotyping of disorders could in turn result in better diagnostics and therapeutics (Insel 2014). Despite the high heritability estimates for most major psychiatric disorders, including attention deficit hyperactivity disorder (ADHD), autism spectrum disorders (ASD), bipolar disorder and schizophrenia (Committee 2009), there are still few genetic markers that reliably associate with these disorders and limited significant genome-wide associations have been documented to date (Sullivan 2011). One possible reason for this is that psychiatric disorders are often heterogeneous at the symptom level and thus uncertainties about phenotype definition may have impeded the discovery of associated genetic risk factors (McLoughlin et al. 2013). A current goal for research in psychiatry is to close the gap in understanding between the symptoms and causes of psychopathology, and move beyond subjective and variable clinical diagnoses to classify disorders based on identifiable neural circuits, and further to link activity in these circuits to the cellular and genetic levels (Insel and Cuthbert 2009).

Within psychiatry, the concept of an endophenotype refers to a heritable quantitative trait, either cognitive or neurophysiological, that is more directly related to dysfunction in neural systems than diagnosis, and which therefore facilitates the identification of genetic variants associated with psychopathology (Gottesman and Gould 2003). Ideally an endophenotype should be heritable, associated with disorder diagnosis and be present in unaffected family members (Hawi et al. 2015). Brain dynamic measures known to relate to the pathophysiology of psychiatric illness can direct search for disorder endophenotypes. One strategy for identifying such measures is to examine cognitive and neural dysfunction closely related to core behavioural symptoms.

Cognitive or executive control, the capacity to flexibly direct and allocate resources to a goal by selecting and integrating relevant contextual information, is critical for higher mental abilities (Blasi et al. 2005). Cognitive control is among the most heritable of neurocognitive traits (Anokhin et al. 2004; Friedman et al. 2008; Macare et al. 2014) and deficits in cognitive control are consistently associated with multiple psychiatric illnesses (Laucht et al. 1997a; Millan et al. 2012; Nieoullon 2002; Royall et al. 2002). Thus there has been a focus on genetic approaches to uncover the biological underpinnings of these measures. Converging evidence from pharmacological, neuroimaging and animal studies indicates that dopamine is critical for the efficiency of cognitive control (for a review, see Cools 2008). Specifically, numerous studies suggest that too little or too much dopamine in the cortex, in particular in the prefrontal cortex (PFC), can be disruptive, impacting both brain dynamics and performance on tasks requiring cognitive control (Bruggemann et al. 2013; Laurens et al. 2010; Luu et al. 2004).

Recent evidence indicates that catechol-*O*-methyltransferase (COMT), which has a crucial role in the regulation of dopamine in the PFC, accounts for some individual variability on tests of executive or cognitive control (Dickinson and Elvevåg 2009; Goldberg and Weinberger 2004). The common polymorphism (Val158Met) predicts activity of COMT; homozygosity for the COMT Met allele is associated with a 35–50% decrease in enzymatic activity and dopamine catabolism relative to Val homozygosity, ultimately resulting in increased availability of dopamine in PFC (Biederman et al. 2008; DeYoung et al. 2010; Dickinson and Elvevåg 2009; Lachman et al. 1996; O’Sullivan et al. 2009). Individuals with the homozygous Met allele (and concomitant PFC dopamine enhancement) show improved cognitive control, working memory, and general intelligence as indexed by both performance, and correlated event-related potential (ERP) and functional magnetic resonance imaging (fMRI) measures (Bertolino et al. 2004; Blasi et al. 2005; Cahoy et al. 2008; Diamond et al. 2014; Eroglu and Barres 2010; Freeman and Rowitch 2013; Joober et al. 2002; Wong and Van Tol 2003). However, other studies using cognitive performance, ERP and transcranial direct-current stimulation (tDCS) measures (Plewnia et al. 2013; Stefanis et al. 2004; Taerk et al. 2004; Tsai et al. 2003) have reported no association between COMT and cognitive control, or even that homozygous Met carriers exhibit abnormal cognitive control activity (Gallinat et al. 2003; Kramer et al. 2007).

Another polymorphism important for understanding genetic effects on executive control is the 7-repeat polymorphism of the DRD4 gene, which decreases dopamine transmission at the D4 receptor (Bellgrove et al. 2005; Johnson et al. 2008; Kiphardt 1974). D4 receptor function may play a role in the etiology of both personality traits and psychiatric illness (Ptacek et al. 2011) with a particular relation to ADHD—an association with the 7-repeat allele is the strongest and most consistently replicated molecular genetic finding in the disorder (Banaschewski et al. 2010; Faraone et al. 2001; Li et al. 2006). Participants carrying the 7-repeat allele also have been reported to show poorer performance on tasks of attentional and executive functions and to have smaller attention-related ERP peaks in such tasks (Albrecht et al. 2014; Vogel et al. 2006). However, findings have again been inconsistent. Some studies have reported that the 7-repeat allele is associated with better performance on executive function tasks (Johnson et al. 2008; Swanson et al. 2000), while other studies have found no such differences (Barkley et al. 2006; Konrad et al. 2010).

Disparities between the results of studies investigating the role of COMT and DRD4 in cognitive control may relate to the use of psychiatric populations (Bertolino et al. 2004; Ehlis et al. 2007; Eroglu and Barres 2010; Stefanis et al. 2004; Taerk et al. 2004) or to the effects of other interacting genes (Wishart et al. 2011). Given that the level of dopamine in the cortex has specific effects on cognitive performance (either too much or too little can lead to impairments) (Bruggemann et al. 2013; Laurens et al. 2010; Luu et al. 2004) it is likely that the epistatic interaction between COMT and DRD4 has an impact on dopaminergic influences on cognitive control. Indeed, one recent study showed no impact of the single genes on an ERP-derived measure of response control (the “NoGo anteriorisation” or NGA) (Fallgatter et al. 1997), whereas there was a strong interaction between COMT and DRD4 on NGA amplitude and also on reaction time variability (Takahashi et al. 2011). This interaction effect may not extend to reward-related processing as measured by ERP markers to feedback on gains and losses (Cavanagh et al. 2012).

Another possible reason for not replicating COMT and DRD4 effects in cognitive control tasks is the relative inffectiveness of the measures used to characterise the underlying neurophysiology of the complex cognitive sub-processes (Cools 2008). Cognitive control has a multifactorial nature. Cognitive electrophysiology as well as functional imaging studies using fMRI have demonstrated that it is associated with activity in a network of brain regions, in particular the basal ganglia, prefrontal, and parietal attentional systems including medial frontal and dorsolateral prefrontal cortex, anterior cingulate cortex (ACC), and precuneus (Ogg et al. 2008; Yoon et al. 2008). Indeed there is evidence that the effects of COMT and DRD4 may contribute to anterior-posterior functional connectivity (Jaspar et al. 2014; Liu et al. 2010; Tian et al. 2013) and thus extend beyond the PFC to areas including the dorsal anterior cingulate (Blasi et al. 2005).

However, it should be noted that the associations previously found between COMT, DRD4, and cognitive ERP measures are not exclusive, and certainly do not suggest a one-to-one relation between dopamine-related gene functions and ERP component measures of cognitive control subprocesses at the scalp or source level.

One of the most studied tasks in relation to cognitive control in psychiatry is the continuous performance task (CPT; Dickinson et al. 2008). The cued version of the task (CPT-AX; Doehnert et al. 2010, 2008; McLoughlin et al. 2010, 2011; Valko et al. 2009) has the advantage of measuring two important components of cognitive control: attentional control (how to allocate attentional resources), via responses to ‘Go’ stimuli, and also response inhibition (monitoring performance in face of conflicts),via responses to “NoGo” stimuli. Deficits in both performance and ERP measures for the CPT-AX task, in particular in the amplitudes of the “Go N2/P3” complex and the corresponding “NoGo N2/P3” peaks aligned with response-initiating and response-inhibiting letter presentations, respectively, are consistently and strongly associated with psychiatric illness, including schizophrenia (Holmes et al. 2005; Lee and Park 2006; Salgado-Pineda et al. 2004), ASD (Lundervold et al. 2012; Tye et al. 2014; Wang et al. 2013) and ADHD (Albrecht et al. 2012; Banaschewski et al. 2004; Brandeis et al. 2006; Huang-Pollock et al. 2012; McLoughlin et al. 2010, 2011).

In the current study, we examined the relationship of two key components of cognitive control, namely the relationship of response execution and inhibition during the (CPT-AX) cued continuous performance task to the COMT val158met and DRD4 exon III VNTR polymorphisms. Previous publications based on this data set have already addressed the genetic modulation of motor postprocessing (Bender et al. 2012a), motor response variability (Bender et al. 2015), and visual postprocessing (Bender et al. 2012b) using statistical analysis at the sensor level of scalp ERPs. To more accurately characterise the complex cognitive sub-processes of cognitive control, we here increased the precision of these EEG measures by estimating brain activity at the source via decomposition of the data into maximally independent source processes using independent component analysis (ICA). ICA identifies component processes in the EEG data that are not only temporally near independent but typically also functionally independent in the sense that they exhibit more distinct response patterns to experimental events of interest than do the same measures applied to the raw channel data, consistent with the fact that scalp channel signals each sum potentials volume conducted to the scalp from a a wide variety of relevant and irrelevant brain and non-brain sources (McLoughlin et al. 2013) (Makeig et al. 1996, 1997).

ICA decomposition has been shown to yield event-related measures that share more genetic variance with behaviour than channel-based EEG measures (McLoughlin et al. 2014), to better characterize individual differences in schizophrenia and ADHD symptomology than scalp channel measures (Lenartowicz et al. 2014; McLoughlin et al. 2014; Rissling et al. 2014). Thus, in general ICA decomposition may enable a more precise characterization of spatiotemporally complex cortical network dynamics associated with cognitive control than equivalent measures applied directly to the recorded data channels.

Based on previous findings, we predicted that carriers of the COMT Met/Met allele will demonstrate improved neural function in the cortical source activities related to response execution and inhibition compared to other COMT alleles, and conversely that individuals with the DRD4 7-repeat will show impaired response execution and inhibition. We also consider the impact of the interaction effect between the two polymorphisms on the neural measures of cognitive control.

## Materials and Methods

### Participants

The current data analysis was conducted on the sample of the Mannheim Study of Children at Risk, a prospective longitudinal study of the outcome of early risk factors from infancy into adulthood (Laucht et al. 2000, 1997b). Children born between 1986 and 1988 were recruited from two obstetric and six pediatric hospitals of the Rhine-Neckar Region of Germany. Infants were included consecutively into the study according to a 2-factorial design intended to enrich and to control the risk status of the sample (full details of the sampling procedure have been reported previously Laucht et al. 1997a). As a result, approximately two-thirds of the study sample had experienced obstetric complications such as preterm birth, and about two-thirds of the families had psychosocial adversities such as marital discord or chronic difficulties. The current investigation included 174 healthy adolescents participating in the 15-year assessment for whom both genetic and 64-channel EEG data were available. Of the initial sample of 384 participants, 18 (4.7%) were excluded because of severe handicaps (neurological impairment or IQ/MQ < 70), 28 (7.3%) were drop-outs at age 15, 35 (9.1%) refused to take part in blood sampling or had incomplete genetic data, and from 43 (11.2%) no (or no reliable) EEG data were available. Intelligence had been assessed at the age of 11 years using the Culture Fair Test 20 (Cattell 1960; RH 1987); the motor quotient was determined at age 11 years by a short version of the Body Coordination Test for children KTK (Kiphardt 1974). 65 subjects (16.7%) were excluded from the current analysis due to a current psychiatric DSM-IV diagnosis. 21 subjects of the remaining 195 (10.8%) had to be excluded because they were not right-handed as indicated by a handedness index above + 60 in the Edinburgh Handedness Inventory (RC 1971). All subjects were free of psychoactive medication at the time of the recording and had a corrected visual acuity of 0.8 or higher. The study was approved by the ethics committee of the Medical Faculty of the University of Heidelberg/Mannheim. Written informed consent was obtained from all participants and their parents.

### Task

Participants performed a computerized A-X version of the continuous performance test (CPT) constructed by doubling the number of trials of a common previous multicenter version (Banaschewski et al. 2003; Bender et al. 2007; van Leeuwen et al. 1998). A total of 800 black-colored capital letters were presented on a white background in the center of the computer screen for 150 ms each. The stimulus onset asynchrony (SOA) between letter presentations was 1600 ms. Whenever an ‘A’ was followed by an ‘X’ (i.e., whenever the letter sequence ‘AX’ was presented) subjects were asked to respond with a rapid right-hand button press of their index finger on the response pad. During testing, the ‘A’ was followed by an ‘X’ 80 times and by some other letter (distractor) 80 times; an ‘X’ without a preceding ‘A’ also occurred 80 times. Non-AX distractors were nine other letters of the alphabet (‘B’, ‘C’, ‘D’, ‘E’, ‘F’, ‘G’, ‘H’, ‘J’, ‘L’). Of these, a frequent distractor (‘H’) was presented 160 times and distractors ‘B’, ‘C’, ‘D’, ‘E’, ‘F’, ‘G’, ‘J’, and ‘L’ each 40 times. Here we present results of analysis of ERP responses to Go cue stimuli (‘X’ following an ‘A’) and NoGo cue stimuli (any other letter following an ‘A’).

### EEG Recording and Preprocessing

Continuous 64-channel DC EEG data were recorded using Neuroscan Sympamps amplifiers (Neuroscan Inc., TX, USA). Sintered silver/silver chloride electrodes were positioned in an equidistant electrode cap montage (Easycap, FMS, Germany). Electrode impedances were kept below 10 k Ohm. The vertical electrooculogram (VEOG) was recorded with bipolar reference by electrodes 1 cm below and above the left eye. The horizontal electrooculogram (HEOG) was calculated by bipolar leads F99 and F109 next to the outer canthii. The recording reference was placed near the left mastoid. The sampling rate was 500 Hz per channel. An anti-aliasing low-pass filter with a cut-off frequency of 100 Hz was employed. The visual stimulation was presented by Gentask of the Neuroscan Stim software package. Reaction times were collected from response triggers from the response pad. EEG data processing was performed offline using the EEGLAB toolbox (v11.0.3.1b) (Delorme and Makeig 2004) for MATLAB (R2012a; The Mathworks, Inc., Natick, Massachusetts). Before analysis, the channel signals were re-referenced to average reference. We then applied a 1-Hz high-pass filter. Time points with any channel value larger than 200 μV in absolute value were rejected from the data and excluded from further analysis. In total, based on our EEG data preprocessing, we rejected the 0.03% of trial for the Go condition and 0.04% for the NoGo.

### Genotyping

EDTA anticoagulated venous blood samples were collected. Leukocyte genomic deoxyribonucleic acid (DNA) was isolated with the Qiamp DNA extraction kit. Genotyping of the *COMT* single nucleotide polymorphism (SNP) was completed using TaqMan (SNP) Genotyping Assays. Genotyping of the *DRD4* exon III VNTR polymorphism was performed using polymerase chain reaction according to Lichter et al. (1993; 14). All genotypes were scored independently by two raters who were blind to the presented data. A further 16 subjects were excluded from the genotype analysis due to noisy EEG data. In detail, the following genotype groups were formed: (1) DRD4 genotypes were classified into two groups according to the presence or absence of the 7r allele:7r (75) versus non-7r (132); (2) COMT: Val/Val (N = 41) versus Val/Met (N = 120) versus Met/Met (N = 46); and (3) COMT X DRD4: Val/Val 7r (N = 14) versus Val/Val non-7r (N = 27), Val/Met 7r (N = 45) versus Val/Met non-7r (N = 75), and Met/Met 7r (N = 16) versus Met/Met non-7r (N = 30).

### EEG Data Analysis

We used adaptive mixture ICA (AMICA) (90, 91) to separate the multichannel data for each into maximally instantaneous independent component (IC) processes. Decompositions used the 64 available channels, removing channels determined to have bad contact (those with extended periods of low correlation with neighboring channels). The sampling rate was 500 Hz. Epochs were 2 s, extending from 800 ms pre-stimulus to 1200 ms post-stimulus. We computed an equivalent dipole model for each IC scalp topography using a template four-layer adult boundary element method head model implemented in the DIPFIT toolbox for MATLAB (92). To optimally combine IC information from different participants, we applied a probabilistic multi-subject inference approach, measure projection analysis (MPA) (93), to event-related potential (ERP) waveforms time-locked to Go and NoGo stimuli, respectively.

Measure projection involves projecting the selected measure into a 3-D grid of voxels filling the template brain volume. For each brain-based IC, the measure is then ‘projected’ to voxels within a spherical region centered on the IC equivalent dipole location. MPA then searches the 3-D voxel grid for voxels for which the projected measures at nearby ICs are (1) sufficiently numerous, and (2) exhibit statistically significant consistency. Here, Measure Projection was applied to Go and NoGo stimulus-locked IC ERP waveforms using EEGLAB default parameter values (significance level, p = .01; maximum domain exemplar correlation, r = 0.7). For each voxel, the projected measures of all ICs whose equivalent dipole location is within the smoothing radius are then summed with weights inversely proportional to the distance of the IC equivalent dipole location from the central voxel. At each voxel, the projected measures for all the brain ICs across the subjects are then normalized.

**Fig. 1:**
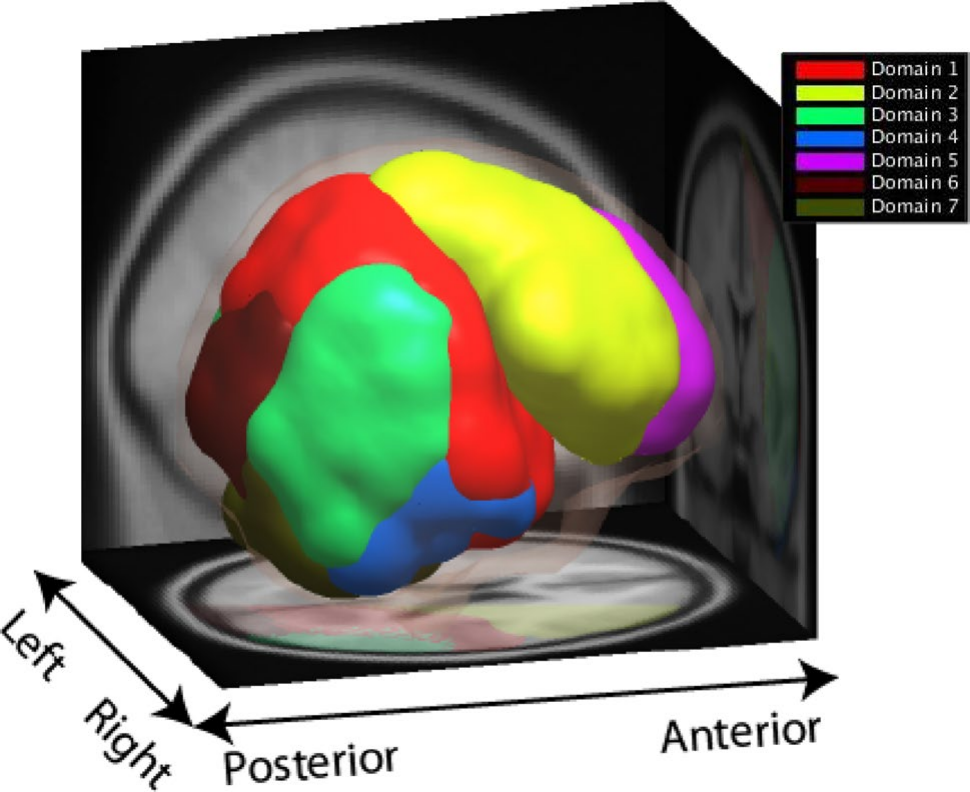
Measure projection domains. We selected the central/parietal Domain 1 (shown in red) and the superior-frontal Domain 2 (shown in yellow) for further analysis as these domains are associated with attention and executive function (Castellanos and Proal 2012; Cortese et al. 2012) and yielded clear N2-P3 type ERPs (the ERPs associated with the other domains did not appear to contribute to the N2 and P3 ERPs)

**Fig. 2:**
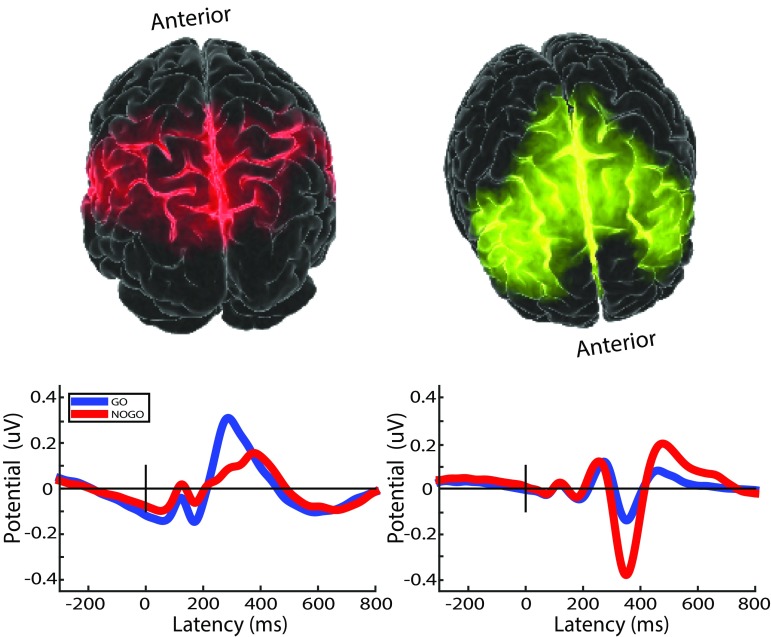
The two selected Measure Projection domains: parietal/temporal cortical source Domain 1 (left, posterior view) and frontal cortex source Domain 2 (right, anterior view) and their associated group grand mean ERP responses to Go and NoGo cue stimuli. ‘P3’ peaks (350–400 ms) were largest in the centro-parietal domain responses, while ‘N2’ peaks (near 350 ms) are largest in the frontal source domain responses. Y-axis unit: peak projected RMS uV across all scalp channels

To simplify the analysis of projected source measure values in the remaining set of voxels (the measure consistency subspace), MPA then separates them into several distinguishable spatial domains using threshold-based affinity propagation clustering as described in detail in (93). Affinity propagation is based on a similarity matrix of pairwise correlations between the projected measures at each voxel position. The method automatically determines an appropriate number of voxel clusters (below referred to as spatial domains) based on the maximum allowed correlation between cluster exemplars, automatically increasing the number of clusters until any other potential cluster exemplar becomes too similar to one of the existing exemplars (93). This approach identified seven spatial domains for the source-resolved ERP data (Fig. 1). Two domains of interest (DOIs) centered in frontal cortex and the parietal/temporal cortex (Domains 1 and 2; Fig. 2) produced prominent P3 and N2 peaks in group grand mean ERPs to Go cue stimuli and were selected for further analysis. ERPs from other identified domains included smaller or ambiguous P3 and N2 peak features and were not considered further.

### Statistical Analyses

For statistical analysis of group differences, genotype groups were compared in terms of peak amplitudes and/or peak area under the curve in trial-average ERPs time-locked to Go and NoGo stimuli where the ‘Go’ stimuli were letter ‘X’ stimuli following letter ‘A’ cue presentations (cueing a button press response), and the NoGo stimuli were any distractor (response-inhibiting) letter following an ‘A’ (i.e., a false alarm condition in which the subject had to inhibit their partially anticipated response). Groups were compared based on their Go and NoGo 200–400 ms post-stimulus ‘P3’ peak amplitudes (Domain 1; Fig. 2). For statistical analysis we used maximal peak amplitude for the Go cue response P3 and the area under the curve for the NoGo cue response P3 as there was no clear peak for the latter. The N2 measure was the maximal negative peak value in the Domain 2 ERP waveforms between 300 and 400 ms post-stimulus (Go and NoGo; Domain 2; Fig. 2). Performance measures were number of commission errors, omission errors, mean reaction time to target stimulus presentations (RT_M_, i.e., mean latency of responding (in ms) following target onsets) and within-subject variability in target reactions times (RT_SD_). We ran multivariate analyses of variance for each ERP measure (Go and NoGo conditions) using Bonferroni correction for posthoc tests. Effect sizes were calculated by converting eta-squared (${\eta }^{2}$) to Cohen’s $d$ using the formula: $d=\sqrt{\frac{{\eta }^{2}}{1-{\eta }^{2}}} \sqrt{2k},$ where $k$ is the number of groups (Cohen 1988).

## Results

### Performance Data

Performance data is presented in Table 1. No main effect of COMT genotype emerged for any of the performance variables [RT_M_: F(1,207) = 0.52, p = .60, d = 0.19; RT_SD_: F(1,207) = 0.60, p = .55, d = 0.17, commission errors: F(1,207) = 0.16, p = .85, d = 0.13; omission errors: F(1,207) = 0.32, p = .73, d = 0.16]. Nor was there any effect of DRD4 genotype on any of the performance variables [RT_M_: F(1,207) = 0.57, p = .45, d = 0; RT_SD_: F(1,207) = 0.15, p = .70, d = 0; commission errors: F(1,207) = 0.83, p = .36, d = 0.08; omission errors: F(1,207) = 0.41, p = .52, d = 0]. Similarly, no interaction effect emerged for any performance variables [NoGo P3; Go N2; NoGo N2; RT_M_, RT_SD_, commission errors; omission errors: all p > .37, all d < 0.25].

**Table 1:** Means and SDs of ERP source variables and cognitive performance variables by each genotype

	COMT Val/Val	COMT Val/Met	COMT Met/Met	DRD4 7-repeat	DRD4 non 7-repeat
Go P3 amplitude	0.34 (0.15)	0.37 (0.18)	0.34 (0.17)	0.34 (0.16)	0.37 (0.17)
NoGo P3 amplitude	0.10 (0.13)	0.10 (0.12)	0.05 (0.09)	0.08 (0.12)	0.09 (0.12)
Go N2 amplitude	− 0.19 (0.13)	− 0.18 (0.17)	− 0.21 (0.14)	− 0.21 (0.19)	− 0.18 (0.13)
NoGo N2 amplitude	− 0.39 (0.19)	− 0.46 (0.38)	− 0.45 (0.24)	− 0.43 (0.34)	− 45 (0.33)
RT_M_	356 (62)	353 (59)	344 (46)	348 (61)	354 (55)
RT_SD_	92 (35)	92 (30)	86 (28)	89 (30)	91 (31)
Commission errors	0.0009 (0.003)	0.001 (0.004)	0.0008 (0.003)	0.001 (0.004)	0.009 (0.003)
Omission errors	0.02 (0.02)	0.019 (0.03)	0.02 (0.03)	0.02 (0.03)	0.02 (0.03)

**Table 2:** Mean and SDs of ERP source variables and cognitive performance variables in the interaction between COMT and DRD4 genotypes

	COMT Val/Val	COMT Val/Met	COMT Met/Met
DRD4 7-repeat			
Go P3 amplitude	0.27 (0.10)	0.39 (0.18)	0.27 (0.10)
NoGo P3 amplitude	0.07 (0.17)	0.10 (0.11)	0.03 (0.06)
Go N2 amplitude	− 0.20 (0.15)	− 0.20 (0.21)	− 0.26 (0.18)
NoGo N2 amplitude	− 0.35 (0.27)	− 0.46 (0.39)	− 0.43 (0.25)
RT_M_	366 (80)	344 (63)	341 (30)
RT_SD_	94 (40)	89 (30)	86 (22)
Commission errors	0.0009 (0.003)	0.002 (0.005)	0.0008 (0.003)
Omission errors	0.01 (0.18)	0.02 (0.04)	0.02 (0.03)
DRD4 non 7-repeat			
Go P3 amp	0.38 (0.15)	0.36 (0.18)	0.38 (0.18)
NoGo P3 amp	0.12 (0.10)	0.10 (0.12)	0.06 (0.11)
Go N2 amp	− 0.19 (0.12)	− 0.17 (0.13)	− 0.20 (0.12)
NoGo N2 amp	− 0.41 (0.30)	− 0.46 (0.37)	− 0.46 (0.23)
RT_M_	351 (52)	358 (57)	346 (53)
RT_SD_	90 (34)	93 (30)	86 (31)
Commission errors	0.0009 (0.003)	0.001 (0.003)	0.0008 (0.003)
Omission errors	0.02 (0.02)	0.02 (0.03)	0.02 (0.03)

### ERP ICA Source Findings

ERP data is presented in Table 2.

#### COMT

**Fig. 3:**
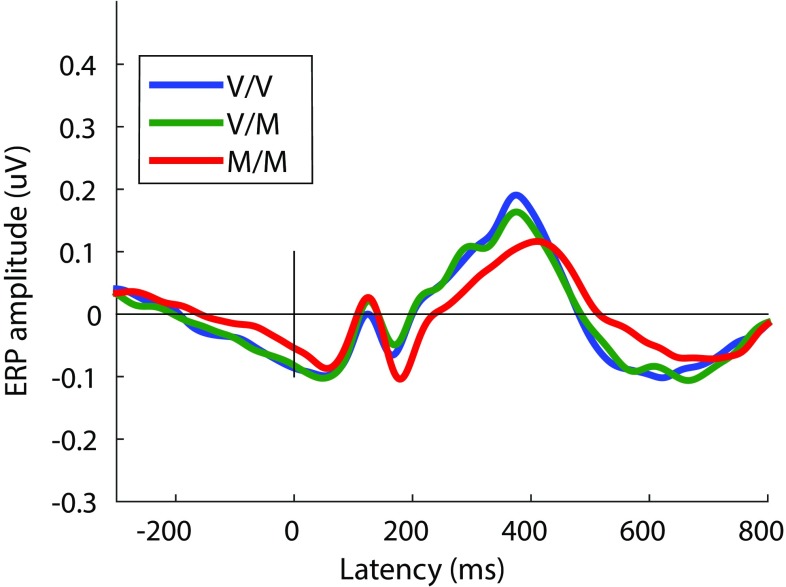
Group grand mean ERP responses to NoGo stimuli for parietal/temporal source Domain 1 (Figs. 1, 2). The P3 peak is the positive-going peak near 400 ms. Y-axis unit: as in Fig. 2. Legend: V/V = Val/Val; V/M = Val/Met; M/M = Met/Met polymorphism groups

**Fig. 4:**
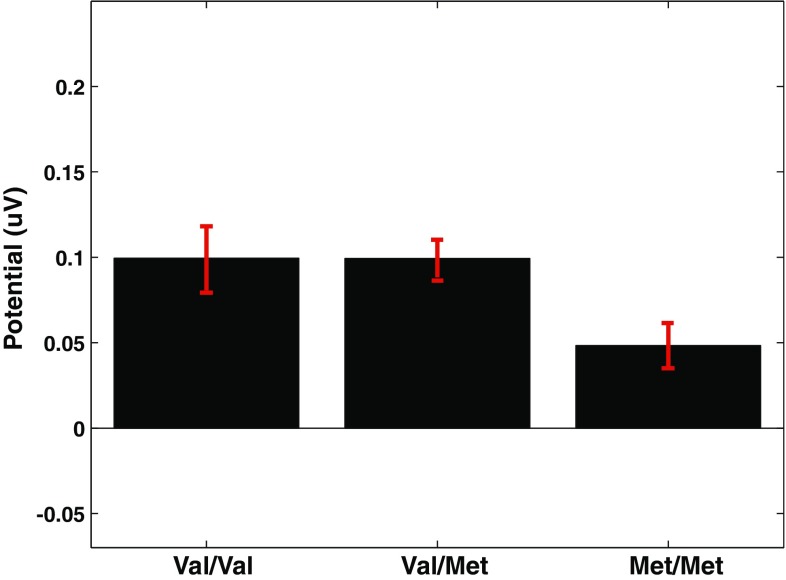
P3 peak amplitudes in ERPs to NoGo stimuli for parietal/temporal source domain (Domain 1) show a main effect of COMT allele. Y-axis unit: as in Fig. 2

A main effect for COMT genotype on P3 amplitude was obtained for the NoGo ERP [F(2, 207) = 3.52, p = .03, d = 0.46] but not for the Go ERP [F(2, 207) = 1.86, p = .16, d = 0.33] (MP domain 1). Posthoc t-tests indicated no difference between the Val/Val and Val/Met groups (p = .10, d = 0) in NoGo P3 amplitude; however the Met/Met genotype group had a significantly smaller NoGo P3 mean amplitude compared to either the Val/Val (p = .04, d = 0.45) or the Val/Met (p = .01, d = 0.44) genotype groups. See Figs. 3 and 4. No main effect emerged for COMT genotype on the N2 amplitude for either condition [Go: (F(2, 207) = 0.66, p = .52, d = 0.19; NoGo: F(2, 207) = 0.71, p = .49, d = 0.46].

#### DRD4

We found a significant effect of DRD4 genotype on Go P3 amplitude [F(2,207) = 5.83, p = .017, d = 0.42], which was smaller in subjects with the DRD4 7-repeat than in those without it. No evidence emerged for an effect of DRD4 genotype on NoGo P3 mean amplitude [NoGo: F(2, 207) = 1.45, p = .23, d = 0.21]. Similarly, no effect emerged for N2 amplitude in either condition [Go: F(2, 207) = 1.48, p = .23, d = 0.17, NoGo: F(2, 207) = 0.150, p = .70, d = 0.11].

#### COMT × DRD4

**Fig. 5:**
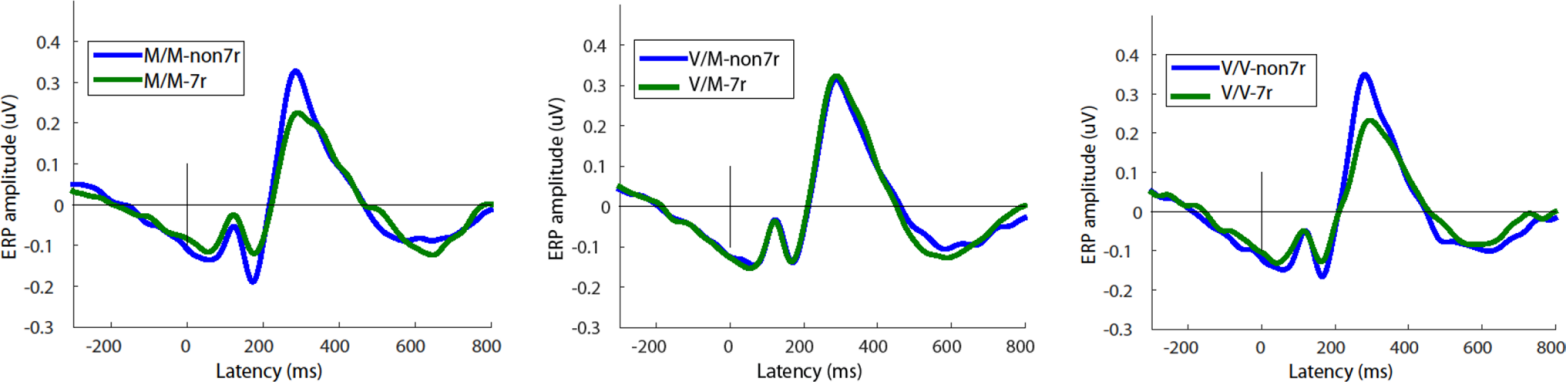
Grand mean group ERP responses to Go cue stimuli for the parietal-temporal source Domain 1 (see Fig. 2) reveal an interaction between COMT and DRD4genotypes on amplitude of the P3 peak. Y-axis unit: as in Fig. 2. *M/M* Met/Met polymorphism; *V/M* Val/Met; *V/V* Val/Val; *non-7r* non 7-repeat; *7r* 7-repeat polymorphism groups

**Fig. 6:**
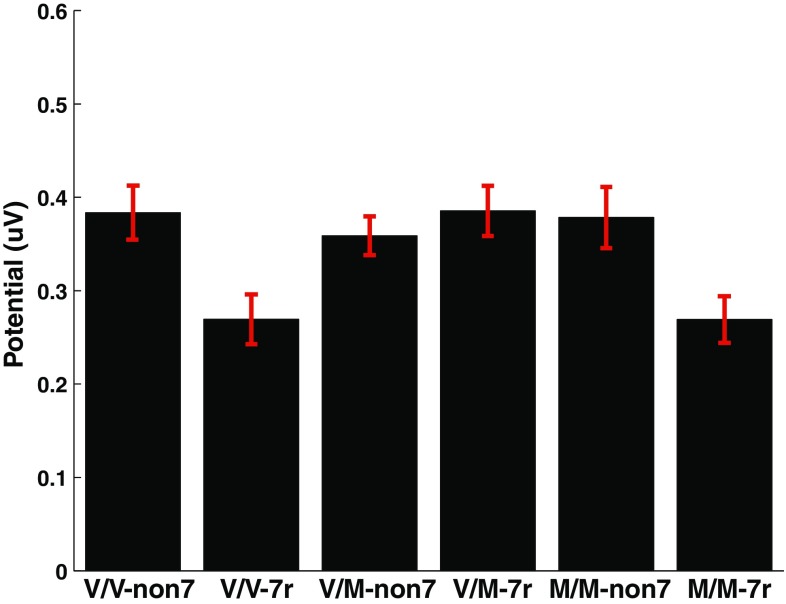
Interaction between COMT and DRD4 alleles on P3 peak amplitude in ERP responses to Go cues. *M/M* Met/Met polymorphism; *V/M* Val/Met; *V/V* Val/Val; *non-7r* non 7-repeat; *7r* 7-repeat

An interaction was detected between the COMT and DRD4 polymorphisms for Go response P3 amplitude [F(2, 207) = 3.99, p = .02, d = 0.49]. *Post hoc* analyses indicated that having the DRD4 genotype reduced Go response P3 amplitude in Val/Val (p = .02, d = 0.80) and Met/Met (p = .03, d = 0.66), but not in Val/Met carriers (p = .43, d=-.14) (Figs. 5, 6).

#### ERP Channel Findings

The same statistical analysis was run on the raw EEG channel data at the scalp channels where the ERP amplitude was maximal. Eye blink components were identified by ICA and removed; the remaining source data were subsequently backprojected to these channels. The amplitudes of the scalp P3 peaks were measured at scalp channel Pz; the N2 peak amplitudes were measured at scalp channel Cz. Similar to the findings at the source level, a main effect for COMT genotype was obtained for Nogo P3 mean amplitude at channel Pz [F(2, 207) = 3.29, p = .04, d = 0.45], but not for Go P3 peak amplitude [F(2, 207) = 2.06, p = .13, d = 0.35]. A significant main effect of DRD4 on P3 peak amplitude at Pz was found for the NoGo condition (F(2, 207) = 6.74, p = .01, d = 0.45) but not for the Go condition (F(2, 207) = 2.74, p = .10, d = 0.28), contrary to the source-resolved findings. No effect of DRD4 on N2 peak amplitude emerged at Cz in either condition [Go: F(2, 207) = 0.41, p = .52, d = 0.11; NoGo: F(2, 207) = 0.82, p = .37, d = 0.16]. No interaction emerged for any of the other channel or performance variables [N2 amplitude at channel Cz in either condition; Go P3; NoGo P3; RT_M_; RT_SD_; commission errors; omission errors: all p > .36, all d < 0.15]. No interaction effects were detected between COMT and DRD4 for any of the channel measures [Go P3 amplitude: NoGo P3; Go N2; NoGo N2; all p > .37, all d < 0.25].

## Discussion

In this targeted candidate gene analysis, we identified several associations between polymorphisms of dopamine system genes and ERP indices of cognitive control during the cued continuous performance task CPT-AX. Specifically, we found that the homozygous Met allele of the COMT genotype was associated with smaller mean P3 amplitude in the IC ERP time-locked to Nogo stimuli and further that individuals with the 7-repeat polymorphism of DRD4 had a smaller peak IC-P3 to Go stimuli. Furthermore, an interaction effect between COMT and DRD4 emerged, indicating a smaller IC-derived Go*-P3* in those with the 7-repeat if they also had the homozygous Val/Val polymorphism *or* the homozygous Met/Met polymorphism, but not in those with the heterozygous Val/Met polymorphism. In contrast to these findings, there was no association between any of the selected polymorphisms and performance measures or the IC-N2 measures.

The overall pattern of task–related ERP activity in our study was consistent with the fronto-parieto midline prominent N2-P3 peak complex observed in tasks that require attentional and cognitive control (Castellanos and Proal 2012; Jaspar et al. 2014). Due to proposed site of action of the COMT enzyme, most of the research on COMT has focused on prefrontally-mediated cognition. Our study instead found that Met allele homozygotes exhibited a smaller ERP measure of response inhibition for a parietal/temporal source domain, but did not identify a significant relationship between COMT and activity in prefrontal cortex. While, COMT alters enzyme activity in prefrontal cortex, and has been strongly associated with activation in frontal brain regions during task performance, the expression of this enzyme is widespread and relatively uniform within the human brain (Hong et al. 2014). Recent findings indicate that the effect of the COMT val^*108*/*158*^met polymorphism extends beyond the PFC and has different effects on brain activity and structure in other regions (Hong et al. 2015), and may specifically include parietal activity during inhibitory control (Van Rooij et al. 2015). These findings indicate that COMT-related inhibitory brain activity is not confined to prefrontal regions.

That the COMT Met/Met allele was associated with an impairment in a neural index of response inhibition may be viewed as surprising, given that the Met allele has been associated with improved performance in executive function and cognitive tests (Cahoy et al. 2008; Freeman and Rowitch 2013; Kiang et al. 2009; Wynn et al. 2010). However, the role of dopamine in the brain and its relationship with the neural network underlying cognitive control is complex (Durstewitz and Seamans 2008; Goldman-Rakic et al. 2000). The dual state or U shaped theories of dopamine regulation (Durstewitz and Seamans 2008; Meyer-Lindenberg and Weinberger 2006) predict that homozygous Met carriers have high tonic dopamine levels in the prefrontal cortex (PFC) and thus optimal levels of dopamine for tasks that require cognitive stability or maintenance, including working memory and competing programs (Nolan et al. 2004), while Val carriers have high phasic dopamine levels in the PFC, and are thus more efficient when cognitive flexibility is required, such as during rapid updating or task switching (Colzato et al. 2010; Drabant et al. 2006; Nolan et al. 2004). Our finding is largely consistent with studies examining the role of COMT that have found that those with the Met/Met polymorphism have impaired response inhibition performance in comparison to Val carriers (Weiss et al. 2014). Our findings are in further agreement with studies that show that the homozygous Met allele is associated with behavioural measures of impulsivity (Soeiro-De-Souza et al. 2013; Stipursky et al. 2011), particularly with a failure to plan ahead (Soeiro-De-Souza et al. 2013) and poorer delayed discounting (Stipursky et al. 2011).

We confirmed our prediction that the DRD4 7-repeat allele was associated with abnormalities in neural indices of attentional control. Previous studies have implicated this polymorphism in attention-related problems in both typically developing children (Auerbach et al. 2001; Schmidt et al. 2002) and in clinical samples of children with ADHD (Faraone et al. 2005). The attention-related EEG activity differences for the parietal/temporal domain in this study are in line with multiple functional imaging studies that showed that attentional control is associated with the activity of a network of brain regions, including the parietal cortex (Buschman and Miller 2007), which has specific involvement in sustained (Coull et al. 1996) and orienting attention (Yantis et al. 2002).

Moreover, we found that the relationship between DRD4 and attentional control (IC-go-P3) varied depending on COMT genotype. This pattern of an epistatic interaction between DRD4 and COMT for attentional control is somewhat consistent with the proposed inverted U-shaped curve for the optimal range of dopamine availability for task performance, according to which too little or too much dopamine is disruptive and impairs functioning of the system (Bruggemann et al. 2013; Congdon et al. 2009; Durstewitz and Seamans 2008; Takahashi et al. 2011). Carriers of the DRD4 7-repeat allele with either of the homozygous COMT polymorphisms presumably had too high levels of dopamine availability for optimal attentional control and exhibited an attenuated pattern of activation to Go stimuli in the CPT-AX. Whereas individuals without the 7-repeat and either of the homozygous COMT polymorphisms may have had optimal levels of dopamine availability. In contrast, there was no influence of the DRD4 7-repeat allele on individuals with the heterozygous Val/Met allele. Dopamine is proposed to modulate the response of neural networks by suppressing spontaneous background firing and thus increasing signal-to-noise ratio and enhancing the task-specific response (Winterer et al. 2006). Our study suggests that both high and low dopaminergic states (Met/Met and Val/Val, respectively) may be compensated by increased D4 function and thus enhancing neural tuning in attentional networks.

A limitation of our study is the candidate gene approach. The selected polymorphisms will not cover all genetic variation within the examined neural circuits. We chose alleles with known functional effects and/or previously reported effects on relevant phenotypes instead of examining a larger set of SNPs tagging the major haplotypes within dopaminergic genes. Therefore, we covered only a small portion of the total dopamine signaling pathway. Already there are many other genes known to be involved in this signaling pathway (Beaulieu and Gainetdinov 2011) and a candidate gene approach will miss most of these signals. Replication is required and the relationship between source localisation measures in EEG and advanced genetic measures, including polygenic scores should be explored.

Despite evidence that psychiatric disorders have genetic etiology (Kendler), there has been a lack of identification of genetic risk factors that reliably associate with psychopathology ({Casey et al. 2013). Psychiatric disorders are likely to involve multiple brain systems and patients may differ in the extent to which processing in these systems is affected so that the biological roots within a clinical diagnosis may vary substantially (Miller 2010). Targeted analysis using functional neuroimaging measures within genetic designs could aid the identification of neural circuits affected in DSM diagnoses to more precisely diagnose and treat psychopathology (Insel and Cuthbert 2015).

No relationship between cognitive performance measures and the genetic markers was found. It may be that the infrequency of required response and inhibition in the CPT-AX (respectively, 10% of stimuli) does not provide enough data points to accurately describe these processes for use in genetic investigations as genetic effects may have relatively small effect sizes compared to, for example, group differences. Genetic effects may be more apparent in information-rich time series data at the neural level than detection via infrequent button presses at the behavioural level. This is reflected in the small effect sizes for all of the cognitive performance measures. Research focusing on overt behavioral correlates of genetic effects on attention and inhibition thus typically uses tasks that require more frequent responding, which may provide greater behavioral resolution of the cognitive process.

Although polymorphisms of COMT and DRD4 had been linked to EEG phenotypes (Loo et al. 2010; Solís-Ortiz et al. 2015), these potential links had not yet been investigated within tasks examining the neurophysiological indices of frontostriatal and parietal attentional networks. Our study aimed to investigate the relationship between COMT and DRD4 polymorphisms and ICA source-resolved brain activity during a cognitive control task, and our findings suggest higher genetic penetrance for IC-derived EEG measures of cognitive control than traditional channel based measures. The associations between these dopamine system gene function and ERP measures to be stronger at the source than at the scalp level, particularly for the COMT x DRD4 interaction; however our results remain correlational. They do not exclude mediating mechanisms, and should not be construed to suggest one-to-one relation between these gene functions and ERP source component measures.

The improved functional and anatomic separation of the cortical signal sources of EEG data produced by ICA decomposition may give measures that may be more informative for genetic studies of brain function. This may be because scalp data channels are each source signal admixtures, so that the effective signal-to-noise ratio of the ICA-separated cortical source activities is under favorable circumstances much higher than for the scalp channel signals (45). This result is consistent with the understanding that for biophysical reasons each scalp channel recording sums activities generated in many places in cortex, whereas many independent component processes separated from the data by ICA decomposition are compatible with an origin in just one cortical area or patch. Thus, neuroimaging methods, including source-based EEG measures, may be more powerful for unravelling gene-brain behavioural relationships than scalp channel-based EEG measures (McLoughlin et al. 2013; Weinberger et al. 2001).

## Figures and Tables

**Fig. 7:**
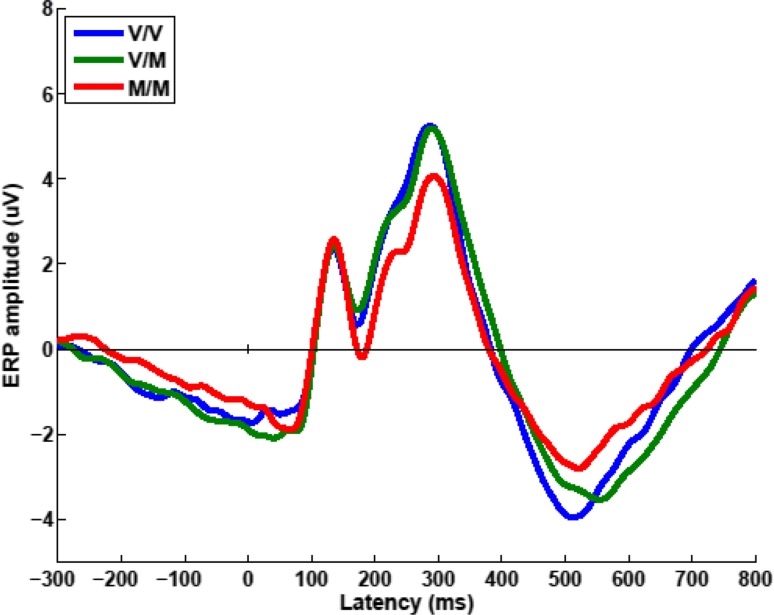
COMT genotype P3 ERP responses to NoGo stimuli at channel Pz. *V/V* Val/Val; *V/M* Val/Met; *M/M* Met/Met polymorphism

**Fig. 8:**
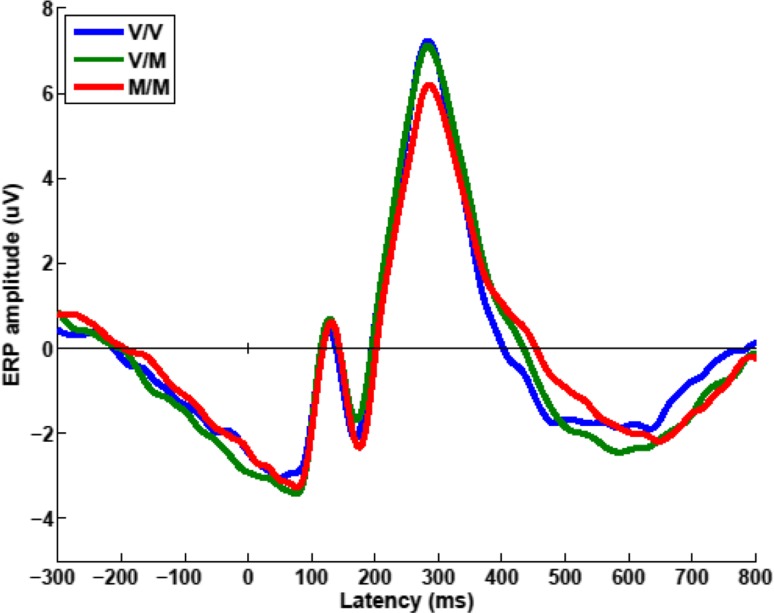
COMT genotype P3 ERP responses to Go stimuli at channel Pz. *V/V* Val/Val; *V/M* Val/Met; *M/M* Met/Met polymorphism

**Fig. 9:**
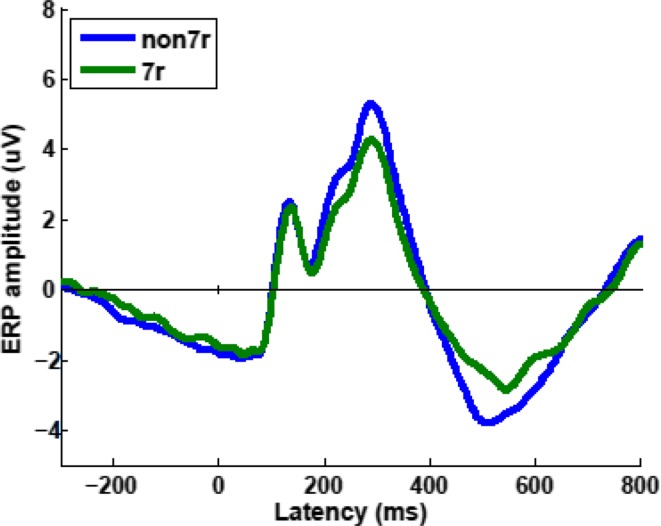
DRD4 genotype P3 ERP responses to NoGo stimuli at channel Pz

**Fig. 10:**
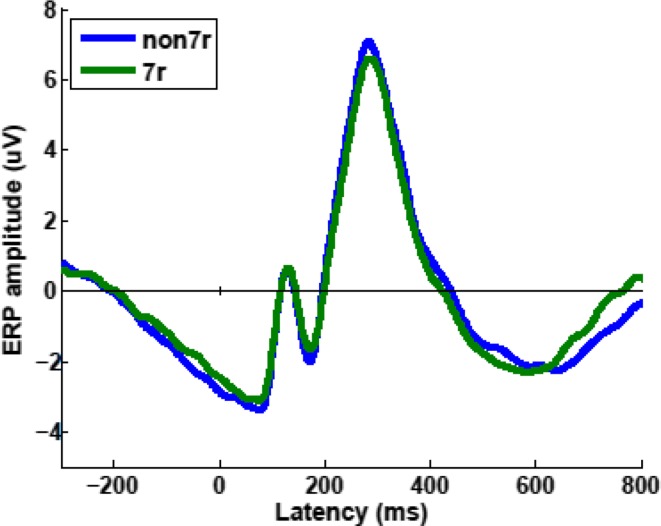
DRD4 genotype P3 ERP responses to Go stimuli at channel Pz

## Appendix

See Tables 3, 4.

**Table 3:** Mean and SDs for ERP scalp variables by genotype

	COMT Val/Val	COMT Val/Met	COMT Met/Met	DRD4 7-repeat	DRD4 Non 7-repeat
Go P3 amplitude (Pz)	6.13 (2.84)	7.01 (2.65)	7.39 (2.51)	6.70 (2.85)	7.04 (2.58)
NoGo P3 amplitude (AUC)	2.29 (2.24)	3.39 (2.56)	3.81 (2.42)	3.11 (2.93)	3.35 (2.25)
Go N2 amplitude (Cz)	− 2.39 (2.81)	− 1.30 (2.40)	− 1.52 (2.98)	− 1.38 (2.86)	− 1.67 (2.52)
NoGo N2 amplitude (Cz)	3.34 (3.02)	8.47 (2.51)	− 3.61 (3.21)	7.36 (2.79)	2.36 (2.83)

**Table 4:** Mean and SDs of the ERP scalp variables and cognitive performance variables in the interaction between COMT and DRD4 genotypes

	COMT Val/Val	COMT Val/Met	COMT Met/Met
DRD4 7-repeat			
Go P3 amplitude	5.73 (2.48)	7.06 (3.06)	6.55 (2.45)
NoGo P3 amplitude	1.93 (1.71)	3.35 (3.28)	3.45 (2.58)
Go N2 amplitude	− 2.15 (2.57)	− 1.23 (2.66)	− 1.12 (3.64)
NoGo N2 amplitude	1.01 (2.53)	1.29 (2.62)	− 1.05 (2.89)
DRD4 non 7-repeat			
Go P3 amplitude	6.34 (3.04)	6.98 (2.39)	7.83 (2.47)
NoGo P3 amplitude	2.47 (2.48)	3.41 (2.05)	3.99 (2.35)
Go N2 amplitude	− 2.52 (2.96)	− 1.34 (2.26)	− 1.74 (2.61)
NoGo N2 amplitude	− 4.71 (3.18)	5.83 (2.42)	5.80 (3.36)

See Figs. 7, 8, 9, 10.
